# Non-Intrusive Cable Fault Diagnosis Based on Inductive Directional Coupling

**DOI:** 10.3390/s18113724

**Published:** 2018-11-01

**Authors:** Suyang Hu, Li Wang, Chuang Gao, Bin Zhang, Zhichan Liu, Shanshui Yang

**Affiliations:** 1 Department of Electrical Engineering, Nanjing University of Aeronautics and Astronautics, Nanjing 210016, China; suyanghu@nuaa.edu.cn (S.H.); chuanggao@nuaa.edu.cn (C.G.); yshanshui@nuaa.edu.cn (S.Y.); 2 Department of Electrical Engineering, University of South Carolina, Columbia, SC 29208, USA; zhangbin@cec.sc.edu (B.Z.); zhichao@ces.sc.edu (Z.L.)

**Keywords:** inductive directional coupling, impedance mismatch, non-intrusive, online diagnosis, signal attenuation, SSTDR

## Abstract

This paper presents and applies an inductive directional coupling technology based on spread spectrum time domain reflectometry (SSTDR) for non-intrusive power cable fault diagnosis. Different from existing capacitive coupling approaches with large signal attenuation, an inductive coupling approach with a capacitive trapper is proposed to restrict the detection signal from transmitting to power source and to eliminate the effect of the power source impedance mismatch. The development, analysis, and implementation of the proposed approach are discussed in detail. A series of simulations and experiments on cables with different fault modes are conducted, along with comparison of existing capacitive coupling, to verify and demonstrate the effectiveness of the proposed method.

## 1. Introduction

Rapid development of complicated large-scale systems, such as aircraft, space stations, nuclear and renewable power systems, brings more and more wires and cables in electrical systems to transmit power and control signals. Stress factors, including water, vibration, and aging [1], will gradually cause degradation of cable conditions and eventually lead to failures, such as open circuits and short circuits. It is desirable to develop a reliable cable diagnosis approach that is able to detect and locate faults as early and accurately as possible, to ensure timely maintenance and reduce unexpected downtime [2].

In the past decades, reflectometry techniques were developed and widely used for cable fault diagnosis [1] because of their advantages in real-time processing and easy design. According to the incident signals, reflectometry methods are divided into Time Domain Reflectometry (TDR) [3,4,5], Frequency Domain Reflectometry (FDR) [6], Sequence Time Domain Reflectometry (STDR) [7,8], Spread Spectrum Time Domain Reflectometry (SSTDR) [7,8], Joint Time-frequency Domain Reflectometry (JTFDR) [9], and Noise Domain Reflectometry (NDR) [10]. Among these methods, NDR, STDR, SSTDR, and JTFDR [11,12,13,14,15] are capable for online diagnosis. Notably, SSTDR has advantages over others in high performance of anti-interference, which means no influence on the normal working of the system.

Although these reflectometry methods show successes in cable fault diagnosis, they are contact techniques that require a detection device connected to the test cable for sending an incident signal and receiving the reflected signal. To deploy these contact cable diagnosis approaches, the existing cable system needs to be disconnected to enable the connection of detection device. This would greatly increase the complexity and cost of deployment and maintenance. To address the limitations of contact approaches, it is desirable to develop non-intrusive cable fault diagnostic approaches. Non-intrusive means to not puncture or remove the protective conducting sheath surrounding the cable, which is ideal from the perspective of safety and infrastructural preservation [16].

Non-intrusive coupling methods can be divided into capacitive coupling and inductive coupling. Non-intrusive inductive coupling is widely used in power line carrier communication, based on the theory of the magnetic coupling [17]. However, there is no reported work showing that the technology has been applied to cable fault diagnosis. Capacitive coupling, on the contrary, has reported works on cable fault diagnosis [18,19,20]. Non-intrusive capacitive coupling, however, has much larger signal attenuation [19], which limits its application to cable fault detection. Different from capacitive coupling, inductive coupling is based on the theory of the magnetic coupling, which makes the inductive coupler simpler and avoids the direct contact of the wire and detection device. This motivates our research on inductive coupling-based non-intrusive approaches.

For both non-intrusive and contact reflectometry techniques, coupled (non-intrusive) and injected (contact) incident signals will transmit in two directions: the power direction and the load direction. Under broadband reflectometry signals, the impedance of the cable is different from that of the power, which leads to false alarms in cable diagnosis [13]. This is an open problem that is not yet addressed in capacitive non-intrusive cable fault diagnosis [19]. In power line carrier communication, high frequency wave trappers are often connected at one end of the coupling device to prevent high-frequency signals from propagating in that direction [21]. However, the device needs to be connected in series and is an intrusive device.

To address the above-mentioned limitations, this paper proposes an inductive directional coupling SSTDR for non-intrusive cable diagnosis. The contributions include: (1) Propose a non-intrusive inductive coupling method that integrates with SSTDR for cable diagnosis; (2) Propose a capacitive trapper in inductive coupling to restrict the detection signal from transmitting to power source and to eliminate the effect of the power source impedance mismatch; (3) Conduct thorough simulations and experimental verifications with comparison to existing approaches to verify the proposed method. Compared with the existing methods, the proposed method has novelties in terms of inductive coupling with lower signal attenuation, and advanced structure for misclassification by impedance mismatch from the power source.

The paper is organized as follows: Section 2 analyzes the theory of inductive directional non-intrusive cable fault diagnosis. Section 3 provides experimental results for verification of the proposed approaches, which is followed by concluding remarks in Section 4.

## 2. Theory of Inductive Directional Non-Intrusive Cable Fault Diagnosis

### 2.1. Theory of Cable Fault Diagnosis by SSTDR

**Figure 1:**
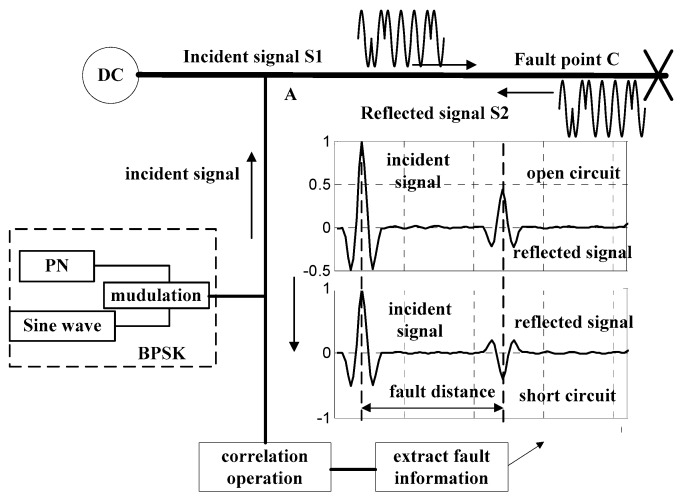
Diagram of Spread Spectrum Time Domain Reflectometry (SSTDR) cable fault diagnosis.

**Figure 2:**
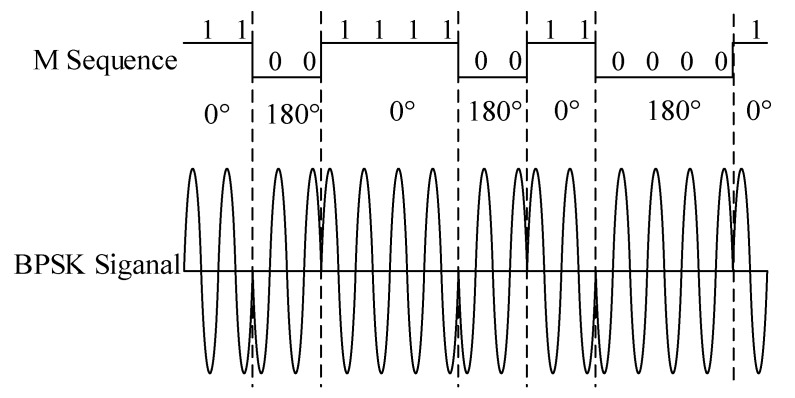
Schematic diagram of m-sequence and sine wave Binary Phase Shift Keyed (BPSK) modulation.

**Figure 3:**
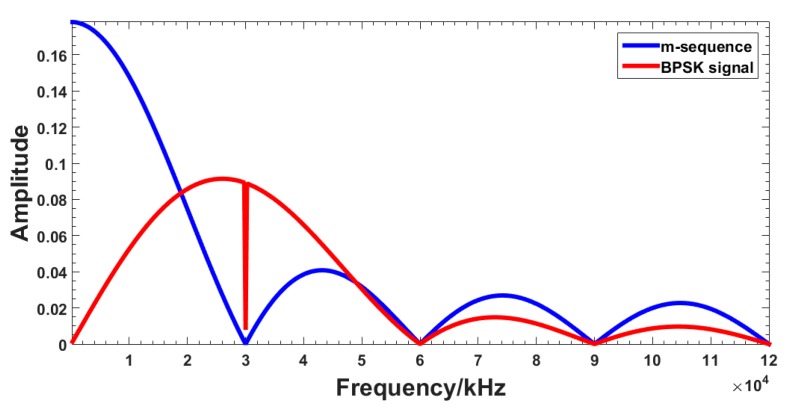
Spectrums of m-sequence and BPSK signal.

Figure 1 shows the diagram of cable fault diagnosis by SSTDR. According to the reflectometry theory, a sine wave is 1:1 modulated by an m-sequence pseudo-noise (PN) code, which results in a binary phase shift keyed (BPSK) signal. The schematic diagram is shown in Figure 2. The spectrums of m-sequence and BPSK signal are shown in Figure 3. It is clear that the energy of the original signal is mainly concentrated within 30 MHz, and the signal spectrum of the modulated BPSK signal is extended to 60 MHz. The modulated signal obtained by this method has strong immunity and correlation. This signal is injected to the cable at the point A. When the signal transmits to fault point C, it is reflected, and the reflected signal is collected at point A. The detection device then calculates the cross correlation of the incident signal and the reflected signal to identify the fault type and location. As shown in Figure 1, the fault type can be determined by the polarity of the reflected signal, relative to the incident signal. The polarity of the open circuit fault is positive and the polarity of the short circuit fault is negative.

The fault location distance *l* can be calculated as

(1) $$l=\frac{1}{2}vt$$

where *v* is the wave propagation velocity and *t* is the time taken for the wave to propagate from the monitoring point (A) to the impedance mismatch point (C), which is then reflected back to point (A). According to the theory of SSTDR, there exists fault location error *d_c_*, which is calculated as:

(2) $$d_{c}=\frac{1}{2}\frac{v}{f_{n}}\;\mathrm{and}\;v=\frac{c}{\sqrt{\varepsilon_{r}}}$$

where *f_n_* is the sampling frequency, *ε_r_* is the dielectric constant of the insulting material, and *c* is the speed of light. Because the cable used in this paper is AF250, the dielectric constant *ε_r_* is equal to 2.08, and the propagation velocity of the incident signal is about 2 × 10^8^ m/s. In this paper, the sampling frequency *f_n_* is 240 MHz, considering hardware cost and performance index. The maximum detection error in the fault location is 42 cm. This method can be applied to other cables by changing the corresponding parameters according to cable types.

### 2.2. Non-Intrusive Inductive Coupling

**Figure 4:**
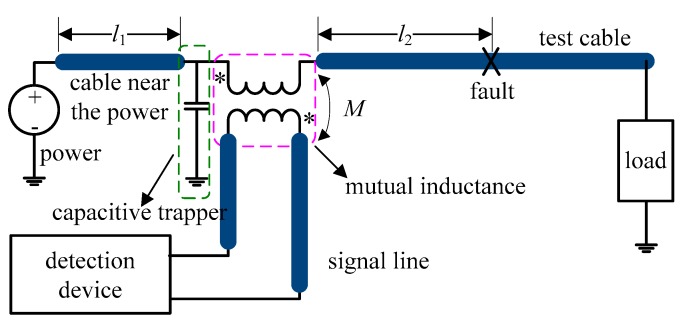
Diagram of non-intrusive inductive coupling cable fault diagnosis, l1 is the distance between the detection device and the power source, l2 is the distance from the detection device to cable fault, M is the mutual inductance of the coupler.

Figure 4 shows the equivalent circuit of non-intrusive inductive coupling cable fault diagnosis. Ferrite cores with high enough relative permeability are inductive couplers, which are installed on the signal line and test cable. Based on the transformer theory, the signal line and the test cable act as the primary side and the secondary side of the transformer, respectively.

Under the effect of the coupler, the incident signal in the signal line is coupled into the test cable. In the same way, the reflected signal in the test cable is coupled into the signal line. Through the incident signal and the coupled reflected signal in the signal line, cable faults can be detected.

**Figure 5:**
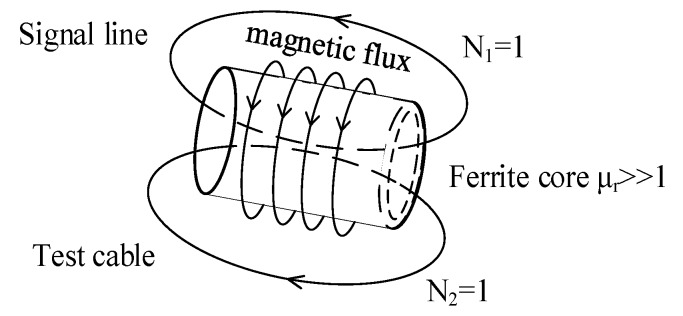
Inductive coupling of two windings.

Figure 5 shows the working principle of the coupler. According to the theory of self and mutual inductance, an electromotive force (emf) is induced in the test cable by the signal line. When the signal line has current *i*_1_, magnetic flux *φ*_11_ is produced. Because of ferrite’s high relative permeability (*μ_r_* >> 1), the magnetic field mainly exists in the ferrite core and almost all magnetic flux is coupled the test cable. 

From the results of finite element simulation, the inductive coupling coefficient is 0.99626 ≈ 1. Then we have,

(3) $$\begin{array}{l} \psi_{11}=N_{1}\phi_{11}=\phi_{11}(N_{1}=1)\\ \psi_{21}=N_{1}\phi_{21}=\phi_{21}\approx \phi_{11}(N_{2}=1) \end{array}$$

where *φ*_11_ and *φ*_21_ are the flux produced by the signal line and the flux of mutual inductance, respectively, *Ψ*_11_ and *Ψ*_21_ are the flux linkage of the signal line and the flux linkage of mutual inductance, respectively, *N*_1_ and *N*_2_ are turns of signal line and test cable, respectively. The inductance of signal line, *L*_1_, and the coefficient of the mutual induction, *M*_21_, are calculated as:

(4) $$L_{1}=\frac{\psi_{11}}{i_{1}}=\frac{\phi_{11}}{i_{1}},M_{21}=\frac{\psi_{21}}{i_{1}}\approx \frac{\phi_{11}}{i_{1}}=L_{1}$$

The inducted emf *u*_21_ of the test cable is:

(5) $$u_{21}=M_{21}\frac{di_{1}}{dt}=L_{1}\frac{di_{1}}{dt}=u_{1}$$

Note that *u*_1_ is the voltage of the signal line. Equation (5) indicates that under ideal cases, the signal in the signal line is successfully coupled into the test cable. Similarly, the reflected signal on the test cable will be coupled into the signal line.

### 2.3. Directional Coupling

As mentioned early, the signal coupled into the test cable transmits to the power source and the load. To address the impedance mismatch of the power source, trappers are used to restrict the transmission of the signal to the power source [13].

In inductive non-intrusive cable fault diagnosis, a capacitive trapper is employed as shown in Figure 4. In the circuit, the capacitive trapper and power source are in parallel. The trapper’s impedance is close to zero for high frequency signals so that the incident and reflected signals do not transmit to the power source. On the other hand, since the impedance of the trapper is very large for low frequency signals, it does not affect the low-frequency or direct current power signals. To deploy the capacitive trapper, copper rings are installed both on the test cable and the ground line. The capacitive trapper is composed of the ring, the cable’s insulation, and the cable’s conductor.

According to the principle of the capacitive trapper, the value of trapper’s impedance is 1/jωC, where ω is the angular frequency of the incident signal and C is the capacitance of the trapper. In this paper, the center frequency of the incident signal is 30 MHz. Theoretically, the trapper should have zero impedance, which cannot be achieved in practice. In this paper, the trapper is designed to make its impedance less than 5 Ω. With this setting, the capacitance value of the trapper must be larger than 1 nF. In this research, the accurate design value of the capacitance of the trapper is determined by trial-and-error in simulations.

**Figure 6:**
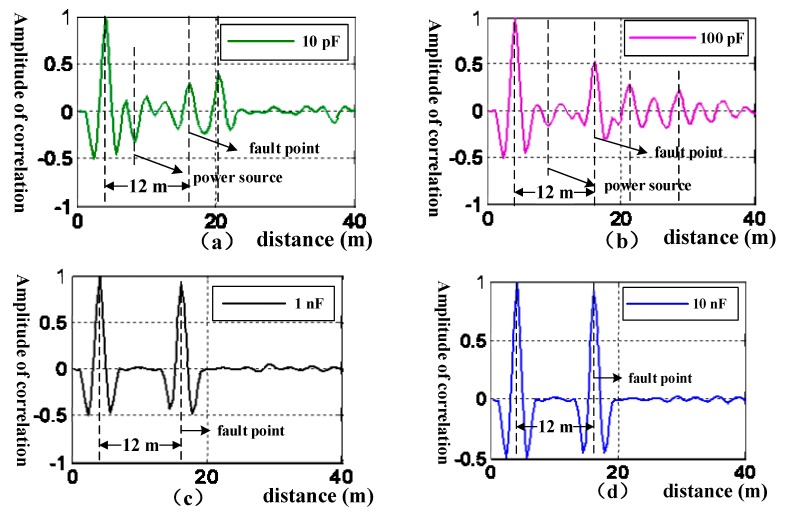
Diagram of cable fault diagnosis with different values of capacitance. (**a**) The trapper with capacitance of 10 pF. (**b**) The trapper with capacitance of 100 pF. (**c**) The trapper with capacitance of 1 nF. (**d**) The trapper with capacitance of 10 nF.

Figure 6 shows the simulation results of cross correlation of the incident and the reflected signals with different capacitance values for the trapper. The simulation is set as follows (see Figure 2): the power is 28 V direct current, *l*_1_ (distance between the detection device and the power source) is 4 m, and *l*_2_ (distance from the detection device to cable fault) is 12 m. Figure 6a,b shows that when the capacitance is smaller than 1 nF, there are reflected waves caused by the impedance mismatch from the power source. The correlation values of the reflected wave from the power source can be larger than that of the reflected wave from the fault point, which can result in misjudgment in diagnosis. On the contrary, when the capacitance is larger than 1 nF, the effect of power source impedance mismatch becomes trivial and the cable fault can be detected correctly, shown as Figure 6c,d.

## 3. Simulation of Inductive Directional Cable Fault Diagnosis

### 3.1. Attenuation Characteristics of Non-Intrusive Coupling Cable Fault Diagnosis

Different from the ideal transformer, parasitic parameters in the inductive coupler will result in attenuation in the process of signal coupling. Too much attenuation will make the amplitude of the reflected signal too small to be detected. To enable fault detection, the attenuation characteristics of inductive coupling needs to be studied. For this purpose, power source impedance mismatch is ignored temporarily to facilitate the analysis.

**Figure 7:**
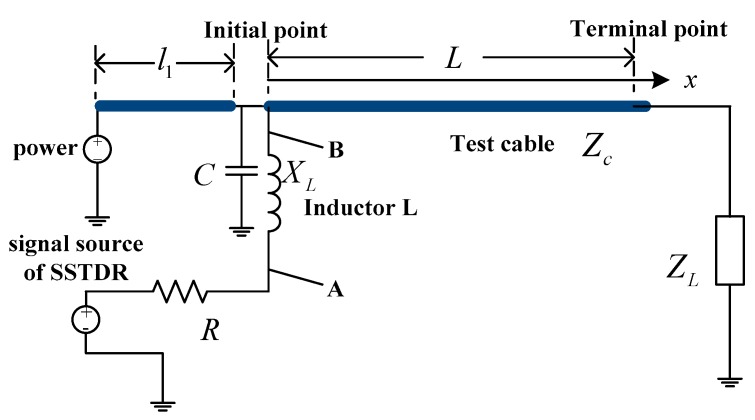
Equivalent circuit of inductive non-intrusive cable fault diagnosis.

Figure 7 shows the equivalent circuit of the inductive coupling non-intrusive cable fault detection system, in which *R* is calculated as 32.09 Ω and *X_L_* as 2.85 Ω. *X_L_* is obtained at the center frequency of 30 MHz.

(6) $$\left\{ \begin{array}{l} u(x)=A_{1}e^{-\gamma x}+A_{2}e^{\gamma x}\\ i(x)=\frac{A_{1}}{Z_{c}}e^{-\gamma x}-\frac{A_{2}}{Z_{c}}e^{\gamma x} \end{array}\right.$$

where *A*_1_ and *A*_2_ are undetermined coefficients, and *γ* is the propagation coefficient, which can be calculated as:

(7) $$\begin{array}{l} A_{1}=\frac{1}{2}(U_{1}+Z_{C}I_{1}),A_{2}=\frac{1}{2}(U_{1}-Z_{C}I_{1})\\ \gamma =\sqrt{\left(R_{0}+jwL_{0})(G_{0}+jwC_{0}\right)}=\alpha +j\beta \end{array}$$

With the source and terminal condition, the voltage of the test cable at a given location *x* from the initial point B can be calculated as follows:

(8) $$u_{\left(x\right)}=\frac{Z_{c}s(t)}{Z_{c}+Z_{g}}\left(\sum_{k=0}^{\infty }{\Gamma_{L}}^{k}{\Gamma_{s}}^{k}e^{-\gamma (2kL+x)}+\sum_{k=1}^{\infty }{\Gamma_{L}}^{k}{\Gamma_{s}}^{k+1}e^{-\gamma (2kL-x)}\right)$$

where Zg = *jX_L_* + *R* and *s*(*t*) is the spread spectrum signal. The characteristic impedance *Zc* keeps constant at high frequency (1 MHz < *f* < 60 MHz). ΓS is the reflection coefficient of the initial point and Γ_*L*_ is the terminal reflection coefficient, which is given as:

(9) $$\Gamma_{s}=\frac{Z_{g}-Z_{c}}{Z_{g}+Z_{c}},\Gamma_{L}=\frac{Z_{L}-Z_{c}}{Z_{L}+Z_{c}}$$

Based on the superposition theorem, the amplitude of signal at point *A*, *S_A_*, can be calculated by the spread spectrum signal *s*(*t*) and the signal amplitude at point *B*, *S_B_*,

(10) $$s_{A}=\frac{jX_{L}}{R+jX_{L}}s(t)+\frac{R}{R+jX_{L}}s_{B}$$

Note that *S_B_* is the voltage signal calculated from Equation (8) with x being equal to zero.

(11) $$s_{A}=\alpha_{0}S\left(t\right)+\alpha_{1}S\left(t-\tau_{1}\right)$$

where *τ*_1_ is the delay time of the reflected signal compared with the incident signal, *α*_0_ is the attenuation coefficient of the incident signal *s*(*t*) in the received signal, and *α*_1_ is the attenuation coefficient of the reflected signal $S\left(t-\tau_{1}\right)$ in the received signal.

The attenuation coefficients *α*_0_ and *α*_1_ can be calculated as follows:

(12) $$\begin{array}{l} \alpha_{0}=\frac{jX_{L}}{R+jX_{L}}+\frac{R}{R+jX_{L}}\frac{Z_{c}}{Z_{c}+R+jX_{L}}\\ \alpha_{1}=\frac{R}{R+jX_{L}}\frac{Z_{c}}{Z_{c}+R+jX_{L}}\times \Gamma_{L}(1+\Gamma_{s})e^{-2\gamma L} \end{array}$$

Correlation of *S_A_* and *s*(*t*) is calculated as:

(13) $$r_{sA}=\int s(t)s_{A}dt=\alpha_{0}\int s(t)s(t)dt+\alpha_{1}\int s(t)s(t-\tau_{1})dt$$

When there is an open circuit in the test cable, Γ_*L*_ = 1, Γ_*S*_ = −0.514, then:

(14) $$\left|\alpha_{0}\right|:\left|\alpha_{1}\right|=1:0.485$$

The reflection coefficient is 0.485. This indicates that the coupled reflected signal is attenuated. Compared with the successful application of SSTDR for open circuits of two AWG 22# wires with reflection coefficient of 0.375 [7], our design has a much higher reflection coefficient and is feasible in the non-intrusive inductive coupling cable fault diagnosis.

To illustrate the advantages of inductive coupling, a comparison of the reflection coefficient with capacitive coupling is given as below. According to Reference [19], the maximum value of the capacitive coupler is 3.0 pF/cm. Suppose that the length of the capacitive coupler is 5 cm, the value of non-contact capacitance is 15 pF. Then, *X_C_* is calculated to be 354 Ω, Γ_*S*_ = −0.87, Γ_*L*_ = 1, and the reflection coefficient of capacitive coupling is:

(15) $$\left|\alpha_{0}\right|:\left|\alpha_{1}\right|=1:\Gamma_{L}(1+\Gamma_{s})\approx 1:0.13$$

The comparison shows that the reflection coefficient of the capacitive coupling is much smaller than that of the inductive coupling. The conclusion is that inductive coupling has much smaller signal attenuation and, therefore, is more suitable for non-intrusive coupling cable fault diagnosis.

### 3.2. Simulation of Inductive Non-Intrusive Coupling Cable Fault Diagnosis

**Figure 8:**
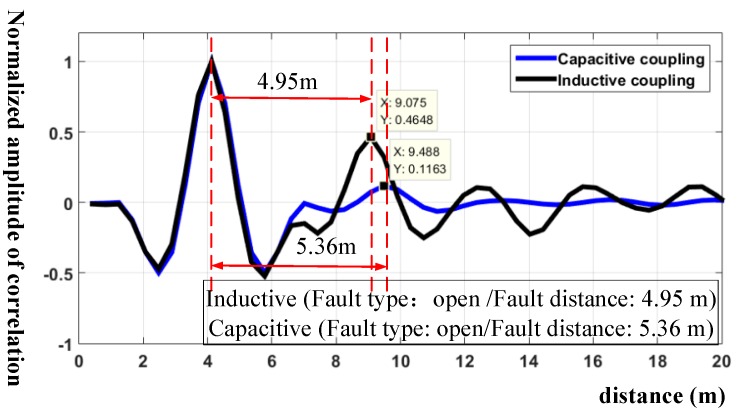
Simulation of cable fault diagnosis with an open circuit at 4.75 m.

For simulation verification, Figure 8 shows the simulation result of detecting an open circuit located at 4.75 m from the initial point.

It shows clearly that the inductive coupling non-intrusive method detects an open circuit located at 4.95 m. The position error is 20 cm, which is in the theoretical error range. The value of the reflection coefficient is 0.465, which is close to theoretical analysis. On the contrary, the capacitive coupling non-intrusive method has a position error of 61 cm, which is larger than the theoretical error. The peak amplitude of the correlation is 0.12, which can easily lead to a false alarm or leak detection. The statistical result of the simulation is shown in the Table 1, which leads to the conclusion that inductive coupling has higher locating accuracy than capacitive coupling. More importantly, the signal attenuation in capacitive coupling is larger than that in inductive coupling, which is consistent with the mathematical analysis and verifies the advantages of the non-intrusive inductive coupling cable fault diagnosis.

## 4. Experiment of Inductive Directional Cable Fault Diagnosis

**Figure 9:**
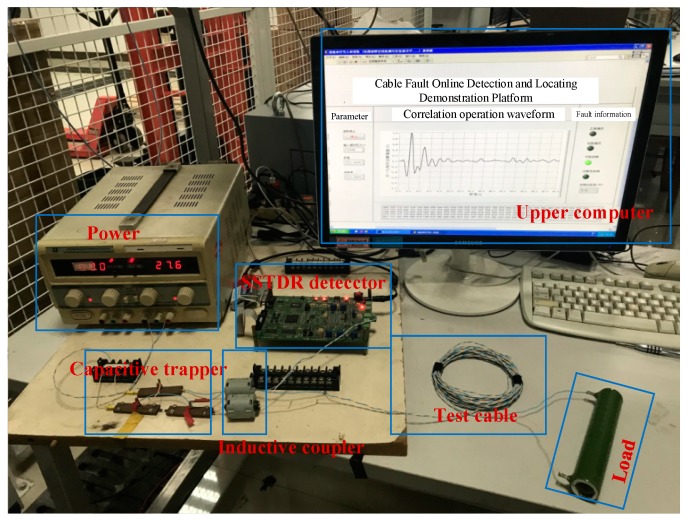
Experimental platform for inductive directional non-intrusive cable fault diagnosis.

**Figure 10:**
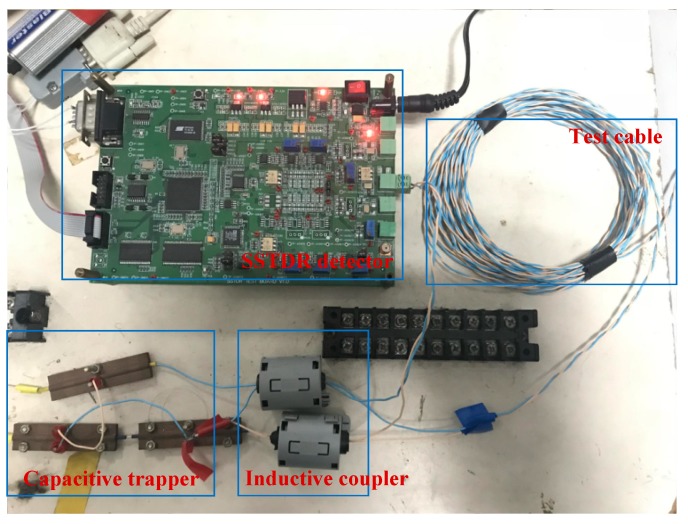
Non-intrusive cable fault diagnosis device.

In this section, experiments of open and short fault modes are conducted to verify the proposed approach. Figure 9 shows the experimental platform, which includes the SSTDR detector, the inductive coupler, the capacitive trapper, the test cable, and the impedance matching networks. The non-intrusive cable fault diagnosis device is clearly shown in Figure 10.

**Figure 11:**
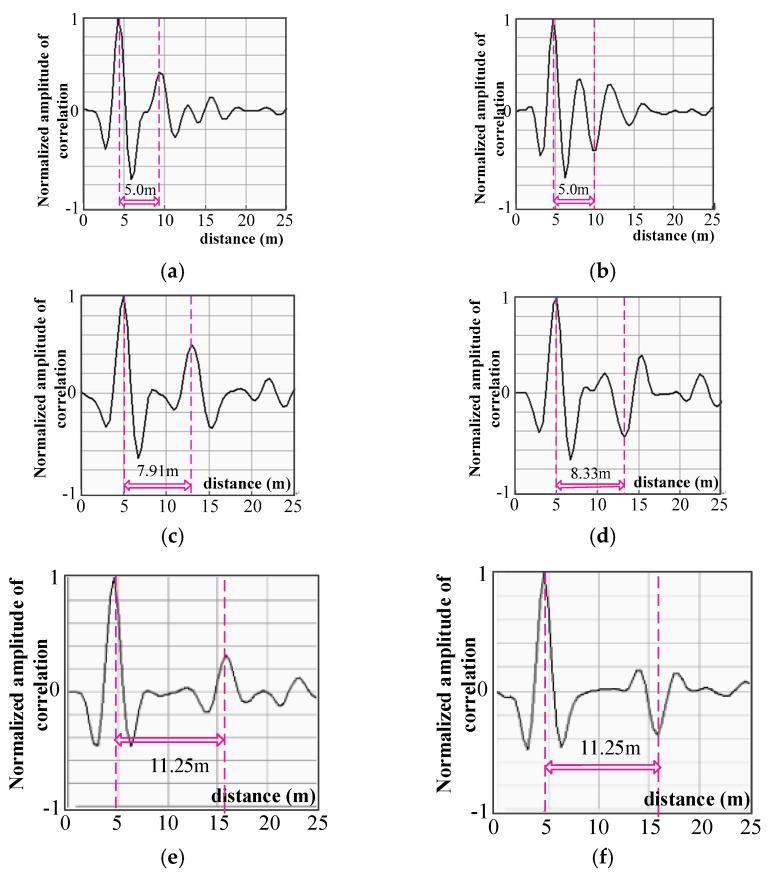
Experimental diagram of inductive directional non-intrusive coupled cable fault diagnosis. (**a**) open circuit of 4.75 m; detected location is 5 m and error is 0.25 m; 167% larger than 2nd largest peaks. (**b**) short circuit of 4.75 m; detected location is 5 m and error is 0.25 m; 66.6% larger than 2nd largest peaks. (**c**) open circuit of 8.10 m; detected location is 7.91 m and error is 0.19 m; 212% larger than 2nd largest peaks. (**d**) short circuit of 8.10 m; detected location is 8.33 m and error is 0.23 m. 156% larger than 2nd largest peaks. (**e**) open circuit of 11.0 m; detected location is 11.25 m and error is 0.25 m; 357% larger than 2nd largest peaks. (**f**) short circuit of 11.0 m; detected location is 11.25 m and error is 0.25 m. 500% larger than 2nd largest peaks.

From the experimental results, it is clear that with the proposed inductive coupling directional non-intrusive cable fault diagnosis, the SSTDR detects fault modes correctly and the detected fault location is in the range of theoretical error. This verifies the feasibility and effectiveness of inductive coupling for non-intrusive cable fault diagnosis. As can be seen from Figure 11, in subfigure (a), the peak at fault location is 167% larger than the 2nd largest peak, the difference between these two peaks is 0.2, and in subfigure (b), the peak at the fault location is 66.6% larger than the 2nd largest peak. In subfigure (c), the corresponding cross-correlation peak is 0.5, which is 212% larger than the 2nd largest peak, with a peak value of 0.16. In subfigure (d), the peak at the fault location is 156% larger than the 2nd largest peak in subfigure (e), which is 357% larger than the 2nd largest peak. In subfigure (f), the peak at the fault location is 500% larger than the 2nd largest peak. More experiments were conducted and the statistical results are summarized in Table 2. In this table, the detection rate is defined as the probability of correct identification in fault samples, which is calculated as:

(16) $$\mathrm{detection}\;\mathrm{rate}=\frac{n}{N}\times 100\%$$

where *n* is the number of samples correctly detected and *N* is the total number of fault samples.

Our conclusion from these results is that the proposed inductive directional non-intrusive coupling cable fault diagnosis method is able to detect both open and short circuits with high accuracy in detecting the fault location.

## 5. Conclusions

In this paper, an integrated non-intrusive cable fault on-line detection method that combines inductive coupling with a capacitive trapper is proposed. The proposed approach improves the feasibility and accuracy of non-intrusive cable fault detection. By adding a capacitive trapper at one side of the coupler, directional transmission of the detection signal is achieved, which solves the issue of detection error caused by power source impedance mismatch. Our contributions include the following findings:

(1) The inductive coupling has the advantage over the capacitive coupling in that it results in small signal attenuation;
(2) For the non-intrusive inductive coupling cable fault diagnosis, although the inductive coupler is needed for signal coupling, the capacitive trappers are required to restrict the detection signal from transmitting to the power source and to eliminate the effect of the power source impedance mismatch.

A series of theoretical analysis, simulations, and experiments are presented for different cable fault scenarios to demonstrate the effectiveness of the proposed approach, which can be extended to other reflectometry-based fault detections. Our future work will focus on non-intrusive online detection of the intermittent cable fault and degradation in insulation.

## Figures and Tables

**Table 1:** Statistical results of the simulation.

Fault Setting		Result of Simulation					
Distance (m)	Type	Capacitive Coupling			Inductive Coupling		
		Type	Distance (m)	Error (m)	Type	Distance (m)	Error (m)
4.75	Open	Open	4.95	0.2	Open	5.36	0.61
5.70	Short	Short	5.75	0.05	Short	5.87	0.17
6.77	Open	Open	6.54	−0.23	Open	7.15	0.35
8.10	Short	Short	8.35	0.25	Short	8.60	0.50
10.14	Open	Open	9.81	−0.33	Open	9.69	−0.45
15.10	Short	Short	14.7	−0.40	Short	14.57	−0.53
20.18	Open	Open	20.03	0.15	Open	20.65	0.47

**Table 2:** Statistical result of experiment.

Fault Setting			Result of Experiment			
Distance (m)	Type	Times	Type	Distance (m)	Error (m)	Detection rate
4.75	Open	100	Open	4.58/5.00	−0.17/0.25	90%
	Short	100	Short	4.58/5.00	−0.17/0.25	90%
5.70	Open	100	Open	5.41/5.83	−0.29/0.13	93%
	Short	100	Short	5.41/5.83	−0.29/0.13	90%
6.77	Open	100	Open	6.66/6.90	−0.11/0.13	93%
	Short	100	Short	6.66/6.90	−0.11/0.13	91%
8.10	Open	100	Open	7.91/8.33	−0.19/0.23	92%
	Short	100	Short	7.91/8.33	−0.19/0.23	91%
10.14	Open	100	Open	9.91/10.33	−0.23/0.19	92%
	Short	100	Short	9.91/10.33	−0.23/0.19	90%
15.10	Open	100	Open	14.9/15.32	−0.2/0.22	91%
	Short	100	Short	14.9/15.32	−0.2/0.22	90%
20.18	Open	100	Open	20.00/20.42	−0.18/0.24	91%
	Short	100	Short	20.00/20.42	−0.18/0.24	92%
